# Performance Modeling of Lightweight Retrieval-Augmented Large Language Models for Low-Resource Plastic Surgery Settings

**DOI:** 10.3390/bioengineering13040378

**Published:** 2026-03-25

**Authors:** Nora Y. Sun, Ariana Genovese, Srinivasagam Prabha, Cesar A. Gomez-Cabello, Syed Ali Haider, Bernardo Collaco, Theophilus Pan, Nadia G. Wood, Antonio Jorge Forte

**Affiliations:** 1 Division of Plastic Surgery, Mayo Clinic, Jacksonville, FL 32224, USA; 2 Department of Statistics, Harvard University, Cambridge, MA 02138, USA; 3 Department of Data Science, University of California, Berkeley, CA 94720, USA; 4 Department of Radiology AI IT, Mayo Clinic, Rochester, MN 55905, USA; 5 Center for Digital Health, Mayo Clinic, Rochester, MN 55905, USA; 6 Department of Artificial Intelligence and Informatics, Mayo Clinic, Jacksonville, FL 32224, USA

**Keywords:** large language models, retrieval-augmented generation, artificial intelligence, plastic surgery

## Abstract

Background: Large language models (LLMs) are being used by surgeons for education and reference yet concerns about hallucinations and reliability limit safe adoption. Retrieval-augmented generation (RAG) can offer a potential solution by grounding responses in a high-quality external database (e.g., medical textbooks) to enhance accuracy. However, performance tradeoffs across different RAG configurations—many of which exponentially increase computational cost—remain poorly characterized. Methods: In total, 120 lightweight, open-source RAG configurations were evaluated across 40 plastic surgery-focused question-answering tasks (20 single-hop, 20 multi-hop), spanning multiple subspecialties (4800 total evaluations). Configurations varied by base LLM (Phi-3-mini-128k-instruct vs. BioMistral-7B), embedding model, database size, chunk size, and query hop type. Performance was assessed using semantic similarity (Ragas) to physician-validated reference answers. Performance was analyzed using linear mixed-effects regression with query as a random effect and fixed and interaction effects selected via likelihood testing and AIC. Results: High performance was achievable using lightweight, open-source models. While BioMistral-7B had high mean sematic similarity under specific configurations (mean semantic similarity up to 0.786), Phi-3-mini-128k-instruct demonstrated more consistent performance across query complexity. Larger database sizes significantly improved semantic similarity, with the largest gain at intermediate sizes (e.g., size 5: +0.043, *p* = 0.001). Embedding choice had a strong effect, with bge-large-en-v1.5 improving performance (*p* = 0.0016) and Bio_ClinicalBERT markedly reducing it (*p* < 0.001). Multi-hop queries substantially reduced performance (*p* < 0.001), though this effect was attenuated for Phi-3-mini-128k-instruct via a strong model × hop-type interaction (*p* < 0.001). Conclusions: RAG systems for plastic surgery do not require large proprietary models, as performance depends on configuration choices and interaction effects rather than isolated components. With advancements, predictive modeling may enable resource-efficient, safe deployment of clinical RAG systems.

## 1. Introduction

### 1.1. Background

Artificial intelligence (AI) tools are entering clinical practice with the promise of expanding clinical insight, decision support, and efficiency [1]. Retrieval-augmented generation (RAG), which grounds large language model (LLM) outputs in external, domain-specific knowledge sources, offers particular promise in healthcare by reducing reliance on parametric memory and potentially mitigating hallucinations that could compromise patient safety [2,3]. This capability is especially important in plastic surgery, where information is dispersed across the extensive and rapidly evolving medical literature [3]. As a result, clinicians frequently consult multiple reference sources during education, planning, and perioperative management.

Despite this potential, there remains a gap between experimental AI systems and tools that can realistically be deployed in clinical environments. Many healthcare institutions operate under substantial computational, financial, and data constraints [4]. Many of the published LLM evaluations in medicine focus on large proprietary models that require significant computational resources or paid API access, limiting their accessibility for real-world clinical use. Consequently, it remains unclear how RAG systems can be configured to achieve reliable performance while remaining feasible for deployment in resource-constrained clinical settings.

At the same time, the number of potential RAG system configurations has grown combinatorially as language models, embedding models, and retrieval strategies continue to evolve. Exhaustive benchmarking of all possible configurations is infeasible due to prohibitive time and computational requirements [5,6]. In response, recent work has shifted away from isolated model comparisons toward approaches that predict LLM performance based on defined sets of model and pipeline parameters [7,8], enabling more efficient design of AI systems.

Several RAG pipeline components such as base LLM selection [9,10], embedding model [9,11], chunk and context size [10,12], database size, and query structure [13] have been shown to influence downstream answer quality. However, many existing RAG-LLM (RALM) studies evaluate the impact of individual parameters in isolation, while holding other variables constant [14]. This approach overlooks the potential for significant and confounding interactions between parameters, which may substantially alter observed performance effects when variables are considered jointly. Understanding these interaction effects is important because design choices—particularly database size—can dramatically affect retrieval latency and cost [15,16].

Although there has been predictive modeling work examining LLM performance across multiple metrics [2], relatively few studies have systematically investigated how RAG configuration parameters affect performance in biomedical contexts. Identifying whether and how these relationships change when multiple parameters are modeled simultaneously is therefore essential to move towards viable deployment.

In addition, the majority of existing benchmarking studies evaluate large (>10 B parameter), closed-source, or proprietary LLMs, which incur substantial financial and computational costs and are often inaccessible to clinics with limited resources [8,17]. In contrast, open-source and lightweight models, which are more compatible with resource-constrained environments, remain underrepresented in biomedical LLM research.

### 1.2. Research Objectives

To address these gaps, we construct a predictive statistical model of retrieval-augmented language model performance on domain-specific plastic surgery question-answering tasks. Rather than benchmarking isolated model configurations, the goal of this study is to examine how RAG pipeline components jointly influence system performance. Specifically, we investigate the association between five variables that influence RAG-LLM performance: four modifiable configuration parameters—base LLM, embedding model, database size, and chunk size—and one task-based property—query hop type. By modeling these factors simultaneously, this study evaluates how configuration interactions influence answer quality across a large set of RAG system configurations.

## 2. Methods

### 2.1. Query Generation

In total, 20 single-hop and 20 multi-hop plastic surgery queries were developed using Claude Sonet 4.

Questions ranged from basic anatomy and physiology (e.g., “What are the names of the two layers of the dermis?”) to complex patient cases (e.g., “A patient with clinically N0 neck has a high-grade mucoepidermoid tumor. What neck treatment should be performed and what structures will be removed”). Topics spanned all subspecialities of plastic surgery, including perioperative management (e.g., “My doctor wants to give me a digital nerve block using both lidocaine and bupivacaine. Why might they mix these medications, and what is the maximum safe concentration of epinephrine for injections in my fingers?”), pediatric surgeries (e.g., “My infant has a facial birthmark that hasn’t gone away by age 3. Given the differences between hemangiomas and vascular malformations, what type of lesion does my child likely have and what treatment approach is indicated?”), and acute trauma and burns (e.g., “How many people die from burn injuries annually in the United States?”). All queries can be found in Supplementary File S1.

Ground truth answers were assigned based on Essentials of Plastic Surgery [18], a comprehensive plastic surgery guide that is well regarded by clinicians. All queries and corresponding reference answers were manually reviewed and refined by two medical doctors to ensure clinical relevance, clarity, and consistency with accepted plastic surgery knowledge with disagreements resolved by consensus. This review process served to verify that the questions and ground truth answers accurately represented established reference material.

### 2.2. Model Configuration

Model configuration and querying was run in Python 3.11. In total, 120 lightweight configurations of 4-bit-quantized RALMs were benchmarked using the 20 single-hop and 20 multi-hop queries (4800 total queries). While benchmarking was run using an A100 GPU through Google Colab to decrease computational time, all configurations can be feasibly queried without GPU access. Configurations tested include every combination of five predictors: base LLM, embedder, database size, chunk size, and hop type.

#### 2.2.1. Base Language Model

Two large language models were utilized for this study: Phi-3-mini-128k-instruct, a general-purpose base LLM, and BioMistral-7B, a domain-specific base LLM.

Both models were selected to reflect the computational and deployment constraints of real-world clinical settings. Each contains fewer than 7 billion parameters, classifying them as lightweight language models relative to contemporary proprietary systems that often exceed 100 billion parameters [19,20]. Lightweight models require substantially fewer computational resources and can be deployed locally without reliance on costly cloud infrastructure [21]. In addition, both models are open source or openly licensed, enabling unrestricted and economical deployment, which is particularly relevant for resource-constrained clinical environments. While there are more recently released biomedical LLMs than BioMistral-7B, they were not lightweight and/or open source and thus were not selected.

The models were chosen to compare generalist and domain-specific language modeling approaches within retrieval-augmented generation pipelines. Phi-3-mini-128k-instruct represents a high-performing small general-purpose model, whereas BioMistral-7B is a widely studied biomedical language model trained on medical text [20,22]. Both models were released recently and are represented among the strongest-performing models within their respective categories [20,23].

#### 2.2.2. Embedder

Three embedding models were evaluated: all-MiniLM-L6-v2, a lightweight general-purpose embedder; bge-large-en-v1.5, a retrieval-optimized embedder commonly used in RAG pipelines; and Bio_ClinicalBERT, a domain-specific biomedical embedder. These models are openly licensed and free to deploy, enabling use in resource-constrained clinical environments. Together, these embedders span generalist, domain-specific, and retrieval-focused design paradigms, allowing comparison of embedding strategies relevant to clinically deployable RAG systems.

#### 2.2.3. Database Size

Ten log_2_-spaced database sizes were tested in this study (Table 1); database size was doubled through addition of new material after each round of experimentation. Tested sizes ranged from 287 128-token chunks, which incorporated less than one-eighth of Essentials of Plastic Surgery [18], to 146,912 128-token chunks, which consisted of twelve scientific textbooks and approximately one-third of the 32-volume Encyclopaedia Britannica [24].

All added database materials were reviewed to ensure that they did not contain information relevant to any queries. This design allowed the database size to increase without introducing additional answer-containing material, enabling isolation of the effect of retrieval scale on system performance. As the database grew, the proportion of irrelevant information increased, simulating the noise and retrieval complexity commonly encountered in large real-world knowledge bases.

#### 2.2.4. Chunk Size

Common chunk sizes include 64, 128, 256, 512, and 1024 tokens [12]. The chunk sizes evaluated in this study were 128 tokens and 256 tokens. These sizes were chosen because larger chunks, such as those with 512 tokens, increase computational cost and noise, while smaller chunks risk loss of meaning [25].

#### 2.2.5. Hop Type

Queries were categorized as either single-hop or multi-hop based on the number of logical reasoning steps required to arrive at a correct answer [20]. Single-hop queries require a single, direct inference and can be answered through straightforward retrieval of factual or descriptive information. For example, single-hop questions that were utilized included: “Why might my doctor choose to use conscious sedation for my operation?”, which requires recalling indications for sedation, and “What are signs of temporal bone trauma”, which requires direct retrieval.

In contrast, multi-hop queries require the integration of multiple pieces of information, such as across anatomical, physiological, or procedural domains. Examples of multi-hop questions included: “During my facial surgery, my surgeon accidentally cuts anterior to the facial artery. Based on nerve-artery relationships and interconnections, which facial nerve branches are at risk and what functional problems might I experience?”, which requires reasoning across anatomical relationships and clinical consequences, and “Which surgical operation and postoperative procedure are needed to restore both facial symmetry and dynamic function in facial nerve palsy?”, which requires combining surgical selection with postoperative management considerations.

Although hop type is not a modifiable property of the retrieval-augmented language model (RALM) configuration itself, evaluating performance across different hop types provides insight into which RAG configurations are better suited for varying levels of clinical reasoning complexity. This distinction is particularly relevant for understanding how RALMs may be optimally deployed across diverse plastic surgery use cases, including factual reference, clinical education, and multi-step decision support.

### 2.3. Performance Evaluation

The semantic similarity metric from Ragas 0.2.15 was the primary metric used to evaluate RALM in this study [26]. Ragas is a suite of tools designed for automated evaluation of RALMs. Semantic similarity was calculated for every generated answer with regard to the ground truth. The semantic similarity metric measures to the extent to which the meaning of the RALM’s answer matches the meaning of the ground truth answer, which better reflects answer quality in comparison to older metrics, such as BLEU [27,28]. Furthermore, semantic similarity is the most widely adapted automated metric with comparison to a ground truth answer that does not rely on prompting another LLM, which may be unreliable [27,29].

### 2.4. Predictive Modeling

All statistical analyses were performed using R version 4.4.2 within RStudio. Preliminary exploration using spline-based model fitting indicated approximately linear relationships between predictors and the outcome variable, supporting the use of linear modeling approaches. Given the relatively small sample size and structured experimental design, no machine learning methods were employed, as such approaches would be prone to overfitting and reduced interpretability. Instead, a regression-based approach was used, so multiple covariates could be considered in the same model while diminishing risk of alpha error due to multiple comparisons.

To evaluate the association between RALM performance and the five study variables, linear mixed-effects regression models were constructed. Mixed-effects models were selected to quantify fixed effects of interest while accounting for sources of random variation that were not themselves meaningful predictors. Because the same set of forty clinical queries was used to evaluate every RAG configuration, each query was assigned an identifier as a random effect to control for query-specific variability in baseline difficulty and noise.

Database size was modeled as a categorical factor with discrete levels, rather than as a continuous variable. Only ten unique database sizes were evaluated in this study, and treating database size as continuous would require assumptions about functional form and extrapolation beyond observed values that are not supported by the experimental design.

Initial linear mixed-effects models without interaction terms were fit separately for each base LLM. These baseline models enabled direct comparison of how individual predictors influenced performance across different base model architectures. Subsequently, a global modeling approach was applied to evaluate interaction effects across all configurations.

To identify appropriate interaction terms, ANOVA was first used to compare a main-effects-only model with a fully saturated model containing all possible interaction terms. Because this comparison demonstrated a statistically significant improvement in model fit, a reduced interaction model was constructed that retained only interaction terms for which at least one component term was statistically significant. This reduced model was then compared with the main-effects model using a likelihood ratio test to confirm improved explanatory power.

Finally, stepwise model selection based on Akaike Information Criterion (AIC) was performed using the lmerTest package to identify the optimal combination of fixed and interaction effects. Cross-validation was not used as mixed-effects modeling includes random effects that would differ between train and test samples; thus, cross-validation is not commonly performed in mixed-effects modeling. To avoid overfitting, AIC implements a penalty for increasing the number of covariates; furthermore, all covariates included in the model were selected *a priori*. This procedure selected the model that achieved the best balance between goodness of fit and model parsimony.

## 3. Results

### 3.1. Overall Performance

Table 2 presents the mean semantic similarity scores for all 120 RAG configurations, ranked from lowest to highest performance. The strongest-performing systems were consistently observed at larger database sizes (typically Size ≥ 6). The single highest-performing configuration used BioMistral-7B paired with the bge-large-en-v1.5 embedder for single-hop queries at database size 10, achieving a mean semantic similarity of 0.786 (95% CI: 0.682–0.889). Other top-ranked configurations clustered in a narrow range between approximately 0.75 and 0.79 and were dominated by BioMistral-7B combined with either bge-large-en-v1.5 or all-MiniLM-L6-v2.

As for the effect of base LLM, BioMistral-7B was used in both the best-performing three configurations and worst-performing two configurations; mean semantic similarity of BioMistral-7B configurations ranged from 0.404 to 0.682. Meanwhile, Phi-3-mini-128k-instruct configurations demonstrated higher consistency, with many scoring between approximately 0.63 and 0.75.

Clear differences were also evident across embedding models. Among configurations in the lower half of Table 2 (mean semantic similarity ≳ 0.60), bge-large-en-v1.5 and all-MiniLM-L6-v2 appeared the most frequently. In contrast, configurations using Bio_ClinicalBERT were largely concentrated in the top half of the rankings, commonly achieving mean semantic similarity values below ~0.55, regardless of base LLM or database size.

Performance patterns differed by query complexity. The three top-performing single-hop configurations used BioMistral-7B, with mean semantic similarity values ranging from 0.75 to 0.786. Meanwhile, the three highest-performing multi-hop configurations used Phi-3-mini-128k-instruct, with mean semantic similarity values ranging from 0.651 to 0.68. Notably, however, confidence intervals overlap for multiple configurations with similar means likely due to smaller samples associated with each specific configuration.

Table 3 presents the performance of Base LLMs by chunk size. 

### 3.2. Combined Mixed-Effects Model

Table 4 presents the results of a combined linear mixed-effects model examining the association between RAG system parameters and semantic similarity. Model coefficients represent the expected change in semantic similarity (on a 0–1 scale) relative to a reference category, holding all other variables constant. The final model included three interaction terms and had improved AIC (−1219 vs. −976) and BIC (−1089 vs. −866) in comparison to the base model. Random-effects variance was 0.020 with a residual variance of 0.043. Residual plots confirmed no significant violations of homoskedasticity and normality. 

Several database sizes were associated with significant improvements in semantic similarity relative to size 1, with the largest gain observed at size 5 (expected change +0.043, *p* = 0.00119). Larger database sizes remained significant (e.g., size 9: +0.034, *p* = 0.01083; size 10: +0.030, *p* = 0.02429), but the magnitude of improvement decreased at higher sizes, suggesting diminishing returns beyond intermediate database sizes.

Effects were also observed for embedding model choice. Relative to all-MiniLM-L6-v2, use of bge-large-en-v1.5 was associated with a significant increase in semantic similarity (expected change +0.023, *p* = 0.00159). In contrast, Bio_ClinicalBERT was associated with a substantial and highly significant decrease in performance (expected change −0.169, *p* < 0.001).

With respect to the base language model, use of Phi-3-mini-128k-instruct, compared to BioMistral-7B, was associated with a significant decrease in semantic similarity in the main effects model (expected change −0.111, *p* < 0.001), prior to accounting for interaction terms.

While chunk size did not show a significant main effect (expected change −0.005, *p* = 0.624), query complexity did exert a strong influence on performance. Multi-hop queries were associated with a large and highly significant decrease in semantic similarity compared to single-hop queries (expected change −0.208, *p* < 0.001).

Several statistically significant interaction effects further clarified these relationships. The interaction between Phi-3-mini-128k-instruct and chunk size 256 was positive and significant (expected change +0.053, *p* < 0.001). Similarly, a strong positive interaction was observed between Phi-3-mini-128k-instruct and multi-hop queries (expected change +0.188, *p* < 0.001), partially offsetting the large negative main effect of multi-hop querying. A smaller but significant positive interaction was also observed between chunk size 256 and multi-hop queries (expected change +0.033, *p* = 0.00540), suggesting modest benefits of larger chunks for more complex query structures.

### 3.3. Main Effects by Base Large Language Model

Table 5 presents mixed-effects-model results examining the relationship between system parameters and semantic similarity for Phi-3-mini-128k-instruct.

Phi-3-mini-128k-instruct demonstrated minimal sensitivity to query hop type. Across all embedders, multi-hop queries were not associated with a significant change in semantic similarity (expected change −0.004, *p* = 0.942). Similarly, no significant hop-type effects were observed within any embedder-specific model (all *p* > 0.05).

In contrast, chunk size had a clear and positive association with performance. Chunk size 256 tokens was associated with a significant overall increase in semantic similarity (expected change +0.065, *p* < 0.001). This benefit was particularly pronounced for configurations using Bio_ClinicalBERT (expected change +0.117, *p* < 0.001) and all-MiniLM-L6-v2 (expected change +0.055, *p* < 0.001). The effect of chunk size was smaller and not statistically significant for bge-large-en-v1.5 (expected change +0.021, *p* = 0.069).

Embedding model selection exerted a strong independent influence on performance. Relative to all-MiniLM-L6-v2, use of bge-large-en-v1.5 was associated with a significant improvement in semantic similarity (expected change +0.029, *p* = 0.003). Bio_ClinicalBERT showed a large and highly significant negative association with performance (expected change −0.178, *p* < 0.001).

Database size exhibited a non-monotonic but qualitatively increasing for Phi-3-mini-128k-instruct. Significant performance improvements were observed at intermediate and larger database sizes, with peaks at approximately 4591 chunks (expected change +0.050, *p* = 0.006) and 73,456 chunks (expected change +0.050, *p* = 0.006). Additional significant gains were observed at 9182, 36,728, and 146,912 chunks, with expected changes ranging from +0.039 to +0.047. Similar patterns were evident in the all-MiniLM-L6-v2 and bge-large-en-v1.5 embedder-specific models, whereas no significant database size effects were observed for Bio_ClinicalBERT.

Table 6 reports mixed-effects-model results examining the association between RAG system parameters and semantic similarity for BioMistral-7B.

In contrast to Phi-3-mini-128k-instruct, BioMistral-7B exhibited strong sensitivity to query hop type. Across all embedders, multi-hop queries were associated with a large and statistically significant decrease in semantic similarity relative to single-hop queries (expected change −0.192, *p* = 0.001). This negative association was consistent across all embedding models, including Bio_ClinicalBERT (expected change −0.195, *p* = 0.002), all-MiniLM-L6-v2 (expected change −0.169, *p* = 0.006), and bge-large-en-v1.5 (expected change −0.212, *p* = 0.001).

Chunk size had a more nuanced relationship with BioMistral-7B performance. In the model pooling across all embedders, increasing chunk size 256 tokens was not significantly associated with performance (expected change +0.012, *p* = 0.145). However, embedder-specific models revealed positive effects of larger chunks for all-MiniLM-L6-v2 (expected change +0.021, *p* = 0.045) and bge-large-en-v1.5 (expected change +0.041, *p* < 0.001). No significant chunk size effects were observed for Bio_ClinicalBERT.

Embedding model choice also influenced performance, though the effect was not always statistically significant. Relative to all-MiniLM-L6-v2, bge-large-en-v1.5 showed a small, marginally positive, but non-significant effect (expected change +0.017, *p* = 0.082). In contrast, Bio_ClinicalBERT was associated with a large and statistically significant decrease in semantic similarity (expected change −0.161, *p* < 0.001), consistent with its poor performance across descriptive and model-based analyses.

Database size effects for BioMistral-7B were selective and embedder-dependent. In the all-embedder model, a significant improvement in semantic similarity was observed at 4591 chunks (expected change +0.037, *p* = 0.037). Within embedder-specific models, all-MiniLM-L6-v2 exhibited significant gains at 4591, 9182, 18,364, and 36,728 chunks, while bge-large-en-v1.5 showed significant improvements across a broader range of intermediate-to-large database sizes, including 4591, 9182, 36,728, 73,456, and 146,912 chunks. As in prior analyses, no significant database size effects were observed for Bio_ClinicalBERT.

**Figure 1:**
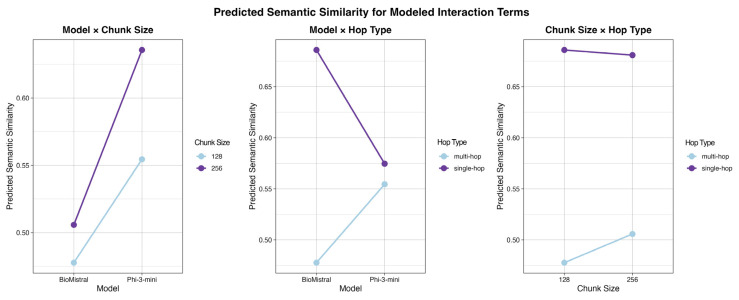
Predicted semantic similarity for modeled interaction terms.

Figure 1 visualizes all statistically significant interaction terms retained after the model selection process as identified in Section 2. 

The interaction between base language model and chunk size reveals that both models benefit from larger chunks, but to markedly different degrees. Phi-3-mini-128k-instruct shows a pronounced improvement in predicted semantic similarity at larger chunk sizes despite having fewer parameters, whereas BioMistral-7B exhibits comparatively modest separation between 128- and 256-token settings.

A second interaction highlights divergent sensitivity to query hop type across models. BioMistral-7B shows a substantial performance gap between single-hop and multi-hop queries, with higher predicted semantic similarity for single-hop questions. In contrast, Phi-3-mini-128k-instruct demonstrates relative robustness to query complexity, with similar predicted performance across single-hop and multi-hop conditions. Table 7 indicates mean semantic similarity by query type and base LLM.

Finally, the interaction between chunk size and hop type indicates that the utility of larger chunks depends on query complexity. For single-hop queries, increasing chunk size yields little to no improvement in predicted semantic similarity and may slightly reduce performance. For multi-hop queries, however, larger chunk sizes are associated with clear gains in predicted performance, suggesting that increased contextual information is particularly beneficial when reasoning over multiple retrieval steps. Table 8 indicates mean semantic similarity by query type and chunk size.

**Figure 2:**
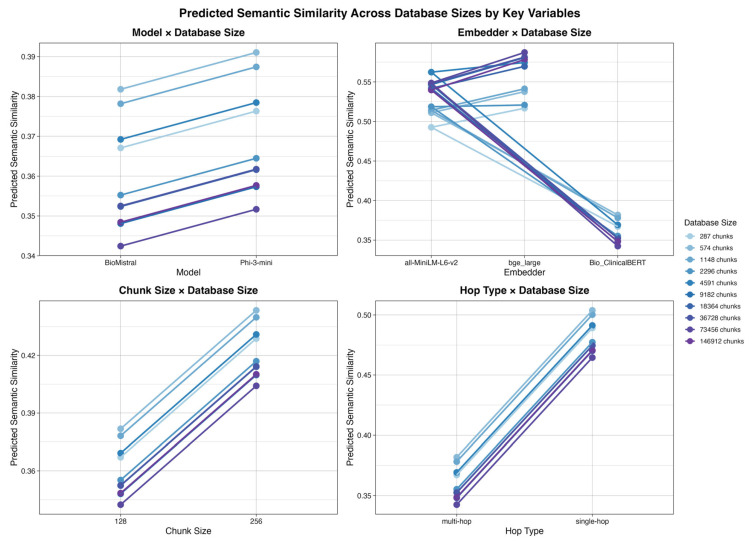
Predicted semantic similarity across database sizes by key variables.

Figure 2 visualizes model-predicted semantic similarity across database sizes, stratified by base language model, embedding model, chunk size, and query hop type.

Across most variables, performance trends as a function of database size were approximately parallel, indicating no significant interaction with database size.

The most visually apparent divergence across database sizes was observed for embedding model choice. Bio_ClinicalBERT consistently exhibited the poorest performance at larger database sizes, while both all-MiniLM-L6-v2 and bge-large-en-v1.5 demonstrated progressive improvements in predicted semantic similarity as database size increased.

Despite this visual separation, the embedder × database size interaction was not statistically significant in the ANOVA comparison between the saturated model and the reduced model without interaction terms. This may be due to small sample size or the modeling of database size as discrete rather than continuous. As a result, embedder × database size was not retained as a statistically significant interaction and does not appear among the interaction effects shown in Figure 1.

## 4. Discussion

### 4.1. Interpretation of the Results

Taken together, these results show that high-performing RAG systems can be achieved in low-resource settings using lightweight, open-source language models paired with general-purpose or retrieval-optimized embedders. The promise of lightweight models is echoed in the literature, with small language model (SML) performance comparable to LLMs [30]. In healthcare settings, lightweight model advantages are particularly consequential, enabling on-premise, privacy-preserving RAG deployment with sub-second latency, substantially lower energy and hardware costs, and improved interpretability for regulatory compliance across resource-constrained environments [31]. While such operational characteristics were not directly evaluated in the present study, these properties motivate interest in lightweight models for resource-constrained clinical environments.

While BioMistral-7B was among the best performers under specific configurations, Phi-3-mini-128k-instruct demonstrated more consistent performance across hop types, reflecting greater robustness to query complexity. This contrast highlights configuration choices, with smaller, generalist models benefiting disproportionately from favorable interactions with chunk size and query structure. This robustness-peak tradeoff mirrors findings in an RAG study where Phi-3-mini-128k-instruct performed as the optimal reader, contributing to the authors’ discussion that larger size does not equate to better performance [32].

Embedding model choice exerted a strong and consistent influence on performance. General-purpose and retrieval-optimized embedders (all-MiniLM-L6-v2 and bge-large-en-v1.5) consistently outperformed the clinically specialized Bio_ClinicalBERT, and domain specialization at the embedding stage did not translate into improved downstream semantic similarity. This may be because Bio_ClinicalBERT was designed for extracting information from clinical notes [33] rather than embedding or retrieval like all-MiniLM-L6-v2 or bge-large-en-v1.5. Furthermore, Bio_ClinicalBERT’s training corpus of clinical notes may differ in many ways to the textbooks used in this study. This may reflect a gap in accessible, domain-specific biomedical embedders for RALMs. However, the embedder choice did interact with database size (Figure 2) and base model behavior (Table 7 and Table 8). Furthermore, prior research on embedding models indicates that they may exhibit unique chunking sensitivities—for instance, some models like Snowflake excel with smaller chunks [12].

Retrieval scale and chunking strategy further shaped performance, but with clear limits. Increasing database size generally improved semantic similarity relative to minimal retrieval, even when additional information was not directly relevant, indicating that broader contextual diversity can support answer generation—a pattern that has been observed across multiple prior RAG studies [34,35]. However, gains peaked at intermediate database sizes and exhibited diminishing returns at larger scales, with the optimal database size differing by base model, suggesting architecture-dependent retrieval capacity. Chunk size effects were conditional, with larger chunk sizes providing benefits for multi-hop queries but little advantage for single-hop queries, reinforcing that context expansion is most useful when query complexity demands integration across multiple retrieval steps. Furthermore, chunk size effects also depended on both the base model and query complexity, as multi-hop queries were consistently more difficult for the models to answer than single-hop queries.

Crucially, RAG performance was governed by interaction effects rather than isolated design choices. Phi-3-mini-128k-instruct benefited substantially more from larger chunks and showed minimal sensitivity to hop type, whereas BioMistral-7B exhibited a large performance gap between single-hop and multi-hop queries. These interactions (Figure 1 and Figure 2) demonstrate that one-size-fits-all RAG configurations are unlikely to be optimal. Instead, predictive statistical modeling provides a principled and scalable alternative to evaluating isolated configurations [8], enabling system designers to align model architecture, retrieval parameters, and expected query complexity with resource constraints when deploying RAG systems in practice.

### 4.2. Clinical Implications

Clinical AI is already being used in practice, largely without formal oversight. Surveys show resident and attending physicians routinely use large language models for learning, diagnostic support, clinical summarization, and documentation [36,37,38]. This informal adoption creates a safety gap: errors produced by AI systems can influence clinical reasoning without transparency or accountability [39]. Regulation and institutional governance have not kept pace [40], leaving patient care exposed to tools whose reliability varies widely and whose failure modes are poorly understood.

Retrieval-augmented generation (RAG) is often proposed as one approach to mitigate these risks by grounding model responses in external knowledge sources [41]. However, RAG systems fail in clinically consequential ways. Prior work has demonstrated vulnerabilities to retrieval failure, noisy or misleading context, and degraded reasoning on multi-step queries [41,42,43,44]. Our results suggest that these behaviors are strongly configuration-dependent. Performance shifts substantially with query complexity, retrieval scale, chunking strategy, and model-retriever alignment, meaning a system that appears reliable under demonstration conditions can behave unpredictably in real clinical use.

Practical clinical use depends not only on answer quality but also on deployability within real-world infrastructure constraints. While larger, domain-specialized models can achieve strong performance under certain configurations, smaller, open-source models paired with retrieval-optimized embedders may provide more stable behavior across varying query structure while requiring fewer computational resources. Because these models are openly licensed and can be run locally, they may be more compatible with resource-constrained environments that cannot rely on proprietary cloud-based systems.

By demonstrating how RAG behavior emerges from interactions among model architecture, retrieval parameters, and query structure, this study reframes RAG as a configurable clinical system rather than a plug-and-play safety solution. The findings therefore represent an early step toward understanding how lightweight RAG systems might be configured for responsible clinical use. However, further work—including correctness evaluation, hallucination analysis, and real-world deployment testing—is necessary before such systems could be considered for clinical implementation.

### 4.3. Ethical Considerations

This study raises important ethical considerations regarding the use of large language models in clinical contexts without adequate oversight. Although the work was conducted in a research setting, its findings are directly relevant to AI tools that clinicians already use informally for learning, documentation, and clinical reasoning. Poorly configured RAG systems can generate confident but incorrect outputs that influence clinical judgment, posing risks to patient safety [41]. Accordingly, this study does not advocate autonomous clinical use and emphasizes AI systems as assistive tools requiring careful validation and oversight.

Equity and access are also central ethical concerns. By demonstrating that high-performing RAG systems can be built using lightweight, open-source components, this work highlights the possibility that clinically relevant AI tools could be developed without reliance on large proprietary systems. Such approaches may improve accessibility for resource-constrained environments that lack the infrastructure or financial resources required to deploy very large models. At the same time, systems that require substantial computational infrastructure risk widening existing disparities in access to clinical AI technologies [45], underscoring the ethical importance of evaluating performance under realistic deployment constraints.

Finally, this study addresses ethical responsibilities related to transparency and governance. RAG is often assumed to reduce risks associated with standalone language models by grounding responses in external reference material [46,47], yet our findings show that retrieval does not eliminate failure modes and can vary depending on configuration. By emphasizing predictive, system-level evaluation rather than ad hoc benchmarking, this work contributes to a more responsible approach to developing clinical AI systems and helps reduce the risk of misplaced trust in automated outputs.

### 4.4. Strengths and Limitations of This Study

A strength of this study is its systematic and statistically rigorous evaluation of RAG system design choices across a large configuration space. By using linear mixed-effects modeling with query included as a random effect, the analysis accounts for query-specific variability and enables principled estimation of both main effects and interaction effects. This approach moves beyond ad hoc benchmarking and provides more generalizable insights into how model architecture, retrieval parameters, and query structure jointly influence performance.

This study is also strengthened by its focus on lightweight, open-source models and resource-constrained configurations, enhancing the relevance of the findings for real-world and clinical deployment. Evaluating both single-hop and multi-hop queries further improves ecological validity by capturing differences in retrieval and reasoning complexity.

Several limitations should be noted. The analysis was restricted to a limited set of base language models, embedding models, and RAG parameters, and the results may not generalize to newer architectures or alternative retrieval strategies. Despite our safeguard of a physician review, the potential for bias from LLM-generated queries must also be noted as a limitation.

In addition, semantic similarity was the sole outcome metric, as a key objective of this study was to develop a predictive model of RALM performance, rather than comprehensively benchmark RALMs across many metrics. Thus, the study did not measure factual accuracy, precision/recall, robustness across query types, and safety-related evaluation, which should be evaluated for these configurations.

Semantic similarity to the physician-reviewed reference answers derived from *Essentials of Plastic Surgery* was used as a scalable measure of reference-answer concordance across 4800 configuration–query evaluations. While this captures overall agreement with validated source material, it does not directly measure faithfulness, hallucination frequency, or clinically important factual errors that semantic similarity does not capture.

This study did not directly model computational cost or latency, limiting conclusions about deployment tradeoffs. Also, this study was performed on an A100 GPU to collect responses to thousands of queries in a timely manner during this experiment. While it is unlikely that any single patient will need to submit this volume of queries, CPU-only deployment of the models should be tested. Future work should explicitly evaluate latency, cost, and real-world deployment. Accordingly, the present study should be interpreted as a feasibility and performance-modeling analysis rather than a safety validation for clinical deployment.

This study is also limited by the configurations, queries, and covariates tested. Database sizes were sampled at discrete intervals, constraining the resolution with which optimal retrieval scale and diminishing returns could be characterized. Additionally, the small number of unique queries limits comparability between configurations, as reflected by the wide 95% confidence intervals for mean semantic similarity. Due to the limited number of queries, only two base LLMs and three embedders were tested in this study.

### 4.5. Future Directions

Future studies should extend this work by evaluating additional healthcare applications [48], base language models, embedding models, and RAG parameters, including newer open-source LLMs, retrieval-optimized embedders, and alternative retrieval or chunking strategies. Expanding the range and resolution of database sizes—particularly with more closely spaced values on a logarithmic scale—and increasing the number of evaluation queries would enable more precise characterization of performance curves and diminishing returns.

In addition to semantic similarity, future evaluations should explicitly incorporate computational cost and resource utilization as outcome variables, framing RAG optimization as a multi-objective problem that better reflects real-world deployment constraints, especially in low-resource and clinical settings. Targeted accuracy and hallucination evaluation should also be conducted to better characterize reliability prior to clinical use.

More broadly, future work should prioritize predictive modeling approaches to better understand RAG system behavior. Given the rapid evolution of LLMs and the limited theoretical understanding of their behavior in retrieval-augmented settings, quantitative models offer a scalable alternative to exhaustive benchmarking. Building on the mixed-effects framework demonstrated here, future studies can estimate system-level effects while accounting for query-specific variability, supporting the development of data-driven guidelines for RAG system design.

## 5. Conclusions

This study demonstrates that the performance and reliability of retrieval-augmented generation systems in plastic surgery are not determined by model choice alone, but by how model architecture, embedding strategy, retrieval scale, chunking, and query structure interact. Using systematic evaluation and mixed-effects modeling across clinically relevant plastic surgery questions, we show that RAG behavior varies substantially across configurations, even when the same base model and retrieval framework are used.

Two findings are particularly relevant for real-world clinical use. First, while BioMistral-7B was among the best performers under optimal conditions, the lightweight general-purpose model Phi-3-mini-128k-instruct demonstrated greater stability across query complexity and configuration choices. Second, general-purpose and retrieval-optimized embedders consistently outperformed a domain-specialized embedder, and increasing retrieval breadth improved performance only up to an architecture-dependent plateau. Peak performance under ideal settings did not reliably predict robustness across clinically realistic, multi-step queries.

By modeling main effects and interaction effects rather than evaluating isolated configurations, this work provides a practical framework for anticipating RAG behavior prior to deployment. For plastic surgery education and decision support, the implication is clear: clinically viable AI depends on system-level design under resource constraints, not on selecting the largest model or most specialized component. Treating RAG systems as configurable clinical tools whose behavior can be predicted and constrained is essential if they are to support clinicians without introducing new risks to patient care.

## Figures and Tables

**Table 1:** Number of 128-token chunks in ten database sizes tested.

Size	Number of 128-Token Chunks
1	287
2	574
3	1148
4	2296
5	4591
6	9182
7	18,364
8	36,728
9	73,456
10	146,912

**Table 2:** Performance of all model configurations at optimal database size ordered by mean semantic similarity.

Base LLM	Embedder	Hop Type	Chunk Size (Tokens)	Optimal Database Size	Mean Semantic Similarity	Lower 95% CI	Upper 95% CI
BioMistral	Bio_ClinicalBERT	Multi-hop	128	Size 2	0.404	0.278	0.531
BioMistral	Bio_ClinicalBERT	Multi-hop	256	Size 3	0.404	0.29	0.519
Phi-3-mini	Bio_ClinicalBERT	Single-Hop	128	Size 10	0.42	0.272	0.568
Phi-3-mini	Bio_ClinicalBERT	Multi-hop	128	Size 5	0.466	0.351	0.582
BioMistral	bge-large-en-v1.5	Multi-hop	128	Size 3	0.523	0.406	0.64
Phi-3-mini	Bio_ClinicalBERT	Single-Hop	256	Size 3	0.529	0.394	0.663
BioMistral	all-MiniLM-L6-v2	Multi-hop	128	Size 7	0.547	0.459	0.635
Phi-3-mini	Bio_ClinicalBERT	Multi-hop	256	Size 1	0.599	0.508	0.689
BioMistral	Bio_ClinicalBERT	Single-Hop	256	Size 3	0.607	0.466	0.748
BioMistral	bge-large-en-v1.5	Multi-hop	256	Size 5	0.615	0.52	0.71
BioMistral	all-MiniLM-L6-v2	Multi-hop	256	Size 5	0.624	0.538	0.709
Phi-3-mini	all-MiniLM-L6-v2	Single-Hop	128	Size 9	0.637	0.52	0.754
BioMistral	Bio_ClinicalBERT	Single-Hop	128	Size 4	0.644	0.533	0.756
Phi-3-mini	all-MiniLM-L6-v2	Multi-hop	128	Size 5	0.65	0.574	0.726
Phi-3-mini	all-MiniLM-L6-v2	Multi-hop	256	Size 9	0.651	0.564	0.738
Phi-3-mini	bge-large-en-v1.5	Multi-hop	128	Size 5	0.67	0.581	0.76
Phi-3-mini	bge-large-en-v1.5	Multi-hop	256	Size 9	0.68	0.599	0.76
Phi-3-mini	bge-large-en-v1.5	Single-Hop	128	Size 8	0.701	0.569	0.833
Phi-3-mini	all-MiniLM-L6-v2	Single-Hop	256	Size 5	0.714	0.581	0.847
BioMistral	all-MiniLM-L6-v2	Single-Hop	256	Size 8	0.728	0.633	0.822
Phi-3-mini	bge-large-en-v1.5	Single-Hop	256	Size 6	0.746	0.645	0.848
BioMistral	all-MiniLM-L6-v2	Single-Hop	128	Size 7	0.75	0.645	0.855
BioMistral	bge-large-en-v1.5	Single-Hop	256	Size 6	0.755	0.674	0.876
BioMistral	bge-large-en-v1.5	Single-Hop	128	Size 10	0.786	0.682	0.889

**Table 3:** Performance of model configurations by chunk size.

Base LLM	Chunk Size (Tokens)	Mean Semantic Similarity	Lower 95% CI	Upper 95% CI
BioMistral	128	0.558	0.513	0.603
Phi-3-mini	128	0.541	0.496	0.585
BioMistral	256	0.569	0.524	0.614
Phi-3-mini	256	0.605	0.560	0.650

**Table 4:** Combined linear mixed-effects model with interaction terms.

Variable	Term	Estimate	*p*-Value
Hop Type	Single-hop (reference)		
	Multi-hop	**−0.208**	**<0.001**
Chunk Size	128 (reference)		
	256	−0.005	0.62437
Embedder	all-MiniLM-L6-v2 (reference)		
	bge-large-en-v1.5	**0.023**	**0.00159**
	Bio_ClinicalBERT	**−0.169**	**<0.001**
Database Size	287 chunks (reference)		
	574 chunks	0.018	0.17595
	1148 chunks	0.019	0.15272
	2296 chunks	0.006	0.64924
	4591 chunks	**0.043**	**0.00119**
	9182 chunks	0.033	0.1323
	18,364 chunks	**0.029**	**0.02869**
	36,728 chunks	**0.035**	**0.00854**
	73,456 chunks	**0.034**	**0.01083**
	146,912 chunks	**0.030**	**0.02429**
Interaction Terms	Model (Phi-3-mini) × Chunk Size (256)	**0.053**	**<0.001**
	Model (Phi-3-mini) × Hop Type (Multi-Hop)	**0.188**	**<0.001**
	Chunk Size (256) × Hop Type (Multi-Hop)	**0.033**	**0.00540**

Significant values (*p* < 0.05) are bolded.

**Table 5:** Phi-3-mini-128k-instruct mixed-effects model: relationship between model performance and predictors across embedders.

Variable	Term	All Embedders		Bio_ClinicalBERT		All-MiniLM-L6-v2		BGE-Large-En-v1.5	
		Estimate	*p*-Value	Estimate	*p*-Value	Estimate	*p*-Value	Estimate	*p*-Value
Hop Type	Single-hop (reference)								
	Multi-hop	−0.004	0.942	0.041	0.379	−0.003	0.955	−0.049	0.411
Chunk Size	128 (reference)								
	256	**0.065**	**<0.001**	**0.117**	**<0.001**	**0.055**	**<0.001**	0.021	0.069
Embedder	all-MiniLM-L6-v2 (reference)								
	bge-large-en-v1.5	**0.029**	**0.003**						
	Bio_ClinicalBERT	**−0.178**	**<0.001**						
Database Size	287 chunks (reference)								
	574 chunks	0.031	0.086	0.018	0.582	0.024	0.337	0.050	0.057
	1148 chunks	0.015	0.389	−0.015	0.647	0.030	0.235	0.031	0.229
	2296 chunks	0.007	0.713	−0.014	0.678	0.030	0.233	0.003	0.902
	4591 chunks	**0.050**	**0.006**	0.014	0.666	**0.081**	**0.002**	**0.054**	**0.039**
	9182 chunks	**0.047**	**0.01**	0.007	0.843	**0.063**	**0.014**	**0.070**	**0.007**
	18,364 chunks	0.028	0.122	−0.021	0.536	0.038	0.139	**0.066**	**0.011**
	36,728 chunks	**0.039**	**0.031**	−0.016	0.631	**0.060**	**0.019**	**0.073**	**0.005**
	73,456 chunks	**0.050**	**0.006**	−0.007	0.84	**0.075**	**0.003**	**0.080**	**0.002**
	146,912 chunks	**0.041**	**0.024**	0.008	0.798	**0.055**	**0.032**	**0.059**	**0.024**

Significant values (*p* < 0.05) are bolded.

**Table 6:** Biomistral-7B mixed-effects model: relationship between model performance and predictors across embedders.

Variable	Term	All Embedders		Bio_ClinicalBERT		All-MiniLM-L6-v2		BGE-Large-En-v1.5	
		Estimate	*p*-Value	Estimate	*p*-Value	Estimate	*p*-Value	Estimate	*p*-Value
Hop Type	Single-hop (reference)								
	Multi-hop	**−0.192**	**0.001**	**−0.195**	**0.002**	**−0.169**	**0.006**	**−0.212**	**0.001**
Chunk Size	128 (reference)								
	256	0.012	0.145	−0.027	0.076	**0.021**	**0.045**	**0.041**	**<0.001**
Embedder	all-MiniLM-L6-v2 (reference)								
	bge-large-en-v1.5	0.017	0.082						
	Bio_ClinicalBERT	**−0.161**	**<0.001**						
Database Size	287 chunks (reference)								
	574 chunks	0.005	0.767	0.011	0.742	0.012	0.591	−0.008	0.752
	1148 chunks	0.023	0.201	0.037	0.273	0.013	0.58	0.018	0.482
	2296 chunks	0.006	0.755	−0.010	0.771	0.022	0.349	0.005	0.848
	4591 chunks	**0.037**	**0.037**	−0.010	0.768	**0.059**	**0.011**	**0.062**	**0.015**
	9182 chunks	0.019	0.27	−0.045	0.192	**0.045**	**0.05**	**0.058**	**0.022**
	18,364 chunks	0.030	0.085	−0.009	0.794	**0.061**	**0.009**	0.040	0.116
	36,728 chunks	0.031	0.077	−0.013	0.697	**0.051**	**0.029**	**0.056**	**0.025**
	73,456 chunks	0.018	0.301	−0.043	0.212	0.037	0.114	**0.061**	**0.016**
	146,912 chunks	0.019	0.273	−0.046	0.18	0.040	0.086	**0.064**	**0.011**

Significant values (*p* < 0.05) are bolded.

**Table 7:** Performance of configurations by base LLM and hop type.

Base LLM	Hop Type	Mean Semantic Similarity	Lower 95% CI	Upper 95% CI
BioMistral	Multi-hop	0.468	0.405	0.530
Phi-3-mini	Multi-hop	0.571	0.509	0.634
BioMistral	Single-hop	0.659	0.597	0.722
Phi-3-mini	Single-hop	0.575	0.512	0.637

**Table 8:** Performance of configurations by chunk size and hop type.

Chunk Size (Tokens)	Hop Type	Mean Semantic Similarity	Lower 95% CI	Upper 95% CI
128	Multi-hop	0.492	0.430	0.555
256	Multi-hop	0.547	0.484	0.609
128	Single-hop	0.606	0.544	0.669
256	Single-hop	0.628	0.565	0.690

## Data Availability

Data is contained within the article or Supplementary Materials.

## Supplementary Materials

The following supporting information can be downloaded at https://www.mdpi.com/article/10.3390/bioengineering13040378/s1, Supplementary File S1: All queries provided to the models.
