# Supplementary material for: Performance Modeling of Lightweight Retrieval-Augmented Large Language Models for Low-Resource Plastic Surgery Settings
Source: Bioengineering (Basel). 2026 Mar 25;13(4):378. doi: 10.3390/bioengineering13040378 (PMC13113923; doi:10.3390/bioengineering13040378)
Supplement: Supplementary file 1 [file bioengineering-13-00378-s001.zip › bioengineering-4163279-supplementary.pdf]

Single Hop Queries

1. What is the active ingredient in Betacaine?
2. Why might my doctor choose to use conscious sedation for my operation?
3. What are signs of temporal bone trauma?
4. On average, how thick is my epidermis?
5. What are the names of the two layers of the dermis?
6. How common is Ehlers-Danlos Syndrome?
7. How many people die from burn injuries annually in the United States?
8. Are residual lesions common in infantile hemangiomas?
9. Can men get breast cancer?
10. Which nerve is responsible for the extrinsic extensor tendons?
11. What numerical score hyperpigmentation correspond to on the Vancouver Scar Scale?
12. What is onion extract and how does it improve scars?
13. According to the American College of Surgeons National Trauma Database, how common is facial injury?
14. What is the difference between the areas of the face that are typically injured during a hands-out fall versus no-hands fall?
15. How much force is required to fracture the facial bone that requires the most force to fracture?
16. What are pediatric considerations for cranialization?
17. What is the most common cause of bilateral facial paralysis?
18. How many grades are in the House-Brackmann Scale?
19. How does cross-face nerve grafting work?
20. What are potential neuromuscular causes of Velopharyngeal Dysfunction?

Multi-Hop Queries

1. My doctor wants to give me a digital nerve block using both lidocaine and bupivacaine. Why might they mix these medications, and what is the maximum safe concentration of epinephrine for injections in my fingers?
2. When advised to undergo flexor tendon repair, what is the optimal timing for my procedure and what postoperative strategy should be employed to minimize adhesions and joint stiffness?
3. During my facial surgery, my surgeon accidentally cuts anterior to the facial artery. Based on nerve-artery relationships and interconnections, which facial nerve branches are at risk and what functional problems might I experience?
4. I'm having minor elective surgery and am opioid-naive. Is opioid reliance very common after these operations, and what protocols can help decrease opioid reliance?
5. My infant has a facial birthmark that hasn't gone away by age 3. Given the differences between hemangiomas and vascular malformations, what type of lesion does my child likely have and what treatment approach is indicated?
6. Which surgical operation and postoperative procedure are needed to restore both facial symmetry and dynamic function in facial nerve palsy?

7. I am worried about developing head and neck cancer. What tends to cause these cancers and what is the main factor determining if I would survive?
8. The doctor said my daughter may have Velopharyngeal Dysfunction, which requires surgery. How do you diagnose someone with Velopharyngeal Dysfunction, and are there any severe potential complications?
9. I have an itchy scar that is thick and raised above the skin. I recently heard of a new treatment that promises to get rid of it by re-aligning collagen fibers at the scar site. Is this strategy likely to work?
10. A patient presents with gynecomastia and small, firm testes. What additional tests should be ordered?
11. A patient on MAO inhibitor therapy requires anesthesia for surgery. Given the need for cardiovascular stability, which anesthetic agent should be avoided and which should be preferred?
12. A patient with both hepatic and renal failure requires rapid sequence intubation but is at risk for malignant hyperthermia. Which neuromuscular blocking agent should be chosen and why?
13. An infant with a hemangioma causing visual obstruction needs treatment, but the parents are concerned about using medications. What are some implications if a more conservative management strategy is chosen?
14. A child presents with limb overgrowth and vascular malformations. Which most likely two syndromes should be considered in the differential diagnosis?
15. A child with syndactyly of multiple fingers needs surgery, but the parents want all procedures done simultaneously. What determines the surgical sequence?
16. A child with syndactyly affecting the third webspace also has craniosynostosis. What surgical expertise is required to treat this child?
17. A patient receiving epidural anesthesia for abdominal wall reconstruction develops plateau pressures of 8 cmH2O but has a history of COPD. What ventilation decision should be made and why?
18. A breast reconstruction patient is concerned about opioid addiction risk but worried about adequate pain control. What approach addresses both concerns according to ERAS recommendations?
19. A patient with facial burns and is developing extensive edema but has normal chest radiographic findings. What urgent evaluation is needed and why?
20. A patient with clinically N0 neck has a high-grade mucoepidermoid tumor. What neck treatment should be performed and what structures will be removed?
